# Message Valence, Industry Influence, and Stakeholder Narratives in Global Conversations on Tobacco Harm Reduction: Content Analysis

**DOI:** 10.2196/77676

**Published:** 2025-11-03

**Authors:** Jungmi Jun, Ali Zain, Minji Kim, James Thrasher

**Affiliations:** 1 School of Journalism and Mass Communications, University of South Carolina, Columbia, SC, United States; 2 Walter Cronkite School of Journalism and Mass Communication, Arizona State University, 555 N Central Ave, Phoenix, AZ, 85004, United States; 3 Arnold School of Public Health, University of South Carolina, Columbia, SC, United States

**Keywords:** tobacco harm reduction, tobacco regulation, public health, social media, stakeholder narrative

## Abstract

**Background:**

Tobacco harm reduction (THR) has become increasingly prominent in global tobacco discourse, with industry actors and advocates actively shaping messaging on social media platforms.

**Objective:**

This study aimed to analyze how THR is discussed on X (formerly known as Twitter), examining message valence toward THR (pro, anti, mixed, or none), stakeholder participation, geographic and temporal variation, and the involvement of industry and THR advocates.

**Methods:**

We conducted a content analysis of 17,361 posts related to THR from 87 countries, published between July 2019 and December 2023. Thematic analysis was used to identify dominant narratives.

**Results:**

Pro-THR posts comprised the majority (12,393/17,361, 71.4%), followed by anti-THR (3925/17,361, 22.6%) and neutral or mixed messages (63/17,361, 0.4%). Pro-THR messages were most prevalent in high-income countries (9193, 78.3%) and were primarily disseminated by THR advocates (7084/7426, 95.4%), tobacco users (3618/3692, 98%), and industry-affiliated accounts (973/1042, 93.3%). Anti-THR posts were more common among government entities (276/333, 82.9%), tobacco control advocates (256/364, 70.3%), and in lower-middle-income regions (149/244, 61.2%). Self-identified health care providers represented 9.4% (1629/17,361) of the dataset, with their posts nearly evenly split between pro-THR (716/1629, 44%) and anti-THR (826/1629, 50.7%) narratives. Pro-THR narratives emphasized the safety and smoking cessation potential of newer nicotine and tobacco products, consumer rights, and skepticism toward public health authorities. In contrast, anti-THR messages focused on youth protection, health risks of newer products, distrust of industry motives, and advocated for complete cessation of tobacco and nicotine use. Notably, 39.6% (6881/17,361) of THR-related posts mentioned newer products, and 15.7% (2724/17,361) included marketing efforts. There was a marked increase over time in overall THR-related post volume, posts by THR advocates, product mentions, and marketing attempts. Overall, high-income countries contributed the majority of posts (11,739/17,361, 67.6%) while nearly half originated from North America (8553/17,361, 49.3%).

**Conclusions:**

The online discourse surrounding THR is characterized by a predominance of pro-THR messaging, particularly in high-income countries and among industry-affiliated stakeholders. The growing volume of THR advocacy and marketing efforts on social media presents new challenges for tobacco regulation and public health policy.

## Introduction

### Background

Tobacco harm reduction (THR) refers to reducing overall health harms, including tobacco-related mortality and morbidity, for both users and the general population, even when exposure to tobacco-related toxicants continues [1,2]. While the complete elimination of tobacco and nicotine exposure would offer the greatest harm reduction, THR advocates acknowledge that complete cessation may not always be possible or desired by users [1,3]. Therefore, THR policies, programs, and practices primarily encourage users to switch to less harmful alternatives from more harmful tobacco products, especially conventional cigarettes [2,4].

The discourse on THR is inherently global, as the associated products and policies transcend national boundaries. Major multinational tobacco companies, including Philip Morris International (PMI), British American Tobacco (BAT), Japan Tobacco International (JTI), and Imperial Brands, develop and distribute newer tobacco and nicotine products, such as electronic nicotine delivery systems (ENDS), heated tobacco products, and oral nicotine pouches across global markets. These companies frequently frame their products using THR-related claims, promoting them as “smoke-free,” “modified or reduced risk,” “healthier,” or “cleaner” substitutes for combustible tobacco [5]. As they introduce newer tobacco and nicotine products (“newer products” hereafter) internationally, the THR messages are globally coordinated and widely disseminated [6].

Social media platforms play a critical role in facilitating the global dissemination of the tobacco industry’s THR claims, ranging from direct product advertising to influencer-based promotions and corporate campaigns [1,7]. Current tobacco control policies and platform restrictions remain inadequate to address the diverse and evolving forms of promotion for newer products as well as their exposure to young people who are active users of social media [8]. Moreover, tobacco companies frequently present THR messaging through corporate social responsibility efforts, news updates, and advocacy framing [9]. All major tobacco firms have launched corporate campaigns reflecting THR messaging, such as PMI’s “Smoke-Free Future,” BAT’s “Building A Better Tomorrow,” JTI’s “Building a Brighter Future,” and Imperial Tobacco’s “Let’s Clear the Smoke” or “Healthier Future.” For example, PMI reported more than 2 million engagements with its #Unsmoke, a theme of the Smoke-Free Future campaign in 2020 [10]. More recently, tobacco companies have sought to amplify the THR discourse to influence scientific narratives and regulatory decisions. These efforts involve the use of lobby and front groups to lend credibility to industry-favorable perspectives while obscuring their origins [11,12]. As a result, global THR messaging on social media not only circumvents traditional advertising restrictions [13] but also potentially undermines the World Health Organization’s Framework Convention on Tobacco Control (WHO FCTC), which explicitly recommends excluding the tobacco industry from policy development [14].

Although THR discourse is increasingly prevalent and the industry’s strategic use of THR messaging continues to grow, there remains limited understanding of how THR is discussed online. Beyond industry voices, a diverse array of influential stakeholders—including governments and regulatory agencies, tobacco control advocates, and scientists—actively shape the global THR conversation as they engage with the public. Analyzing social media discourse surrounding THR can facilitate international cooperation in regulating emerging products, most of which are promoted through THR narratives. Public sentiment is instrumental in shaping tobacco control policies [15], making it essential to understand these perspectives to develop effective, evidence-based regulations that address both local and global health concerns. In addition, THR raises important questions of equity, as countries with a strong tobacco industry presence or significant influence in global regulatory arenas often steer international discussions [16]. This, in turn, affects how THR is perceived and governed in nations with emerging or less-developed regulatory frameworks. By tracking global sentiment and identifying the key stakeholders shaping this narrative, the global health network of tobacco control scientists and advocates can better anticipate, counter, and respond to industry messaging, while fostering a more inclusive and evidence-based dialogue on THR.

Accordingly, this study analyzes posts about THR on X (formerly known as Twitter) from July 2019 to December 2023, focusing on the proportion and key themes of posts expressing positive, negative, neutral, or mixed perspectives on THR. In addition, it identifies stakeholder involvement in the THR discourse, with particular attention to industry representatives and THR advocates. Finally, the study examines regional and temporal variations, including major events associated with shifts in THR-related discourse over time.

THR remains a contentious issue in public health, with governments, experts, and stakeholders expressing a wide range of views globally [3,17,18]. Within the tobacco control community, perspectives range from strong advocacy for THR to deep skepticism. Debates center around key issues, such as (1) the extent of risk reduction offered by newer products, (2) their effectiveness in helping individuals quit more harmful tobacco use, (3) the long-term consequences of nicotine dependence, (4) the appropriate regulation of these products, the role of free-market mechanisms versus collaboration with the tobacco industry, and (5) the challenge of balancing potential risks to youth and nonsmokers with the potential benefits for adult smokers [2].

Compounding these differences, national stances on THR vary significantly. While some countries have embraced THR within their tobacco control strategies, actively promoting some newer products at the national level (eg, UK Department of Health and Social Care) [19], others maintain a more cautious or restrictive approach [20]. Regulatory frameworks also diverge, particularly in how countries classify and oversee newer products marketed with THR claims [21-23]. Public perceptions may differ across regions, as they are shaped by local factors, such as the strength of tobacco control advocacy, susceptibility to industry influence, and the availability of health infrastructure, including education campaigns, health literacy, and smoking cessation programs [18,24]. These varying viewpoints inevitably extend into social media, which serve as critical venues for information sharing and advocacy communications among tobacco control stakeholders [25].

### Message Valence Toward THR

Valence—the positive, negative, or neutral attitude toward a topic expressed in a message—is a critical dimension of communication analysis [26]. Authors may convey valence either intentionally or unintentionally through explicit statements or by selectively presenting information or perspectives. Valence influences not only audiences’ attention and cognitive processing but also their broader attitudes and behaviors related to the topic [27]. Moreover, repeated exposure to messages with a specific valence, especially in public spaces like social media, can shape perceived social norms, such as the sense that most people support or oppose a certain view [28,29].

Valence or sentiment analysis has been widely used in research on tobacco-related social media content. Prior studies have classified sentiment in posts referencing newer products, including electronic cigarettes (e-cigarettes) [30-33], heated tobacco products [34,35], and oral nicotine pouches [36,37]. According to these studies, protobacco or promotional posts consistently outnumbered negative or nonpromotional ones. Sentiment analysis has also been applied to posts about tobacco control policies related to THR, such as US Food and Drug Administration (FDA) authorizations of modified risk tobacco products (MRTPs), revealing more pro-MRTP content than anti-MRTP counterparts [38].

Prior studies also suggest that regulatory announcements are linked to shifts in sentiment online. For instance, negative sentiment spiked following the FDA’s flavored e-cigarette policy announcement [39] and similar regulations in New York State [40]. In this context, social media discourse about THR in terms of both volume and sentiment is likely influenced by significant events, such as the introduction of newer products, industry campaigns, or major policy decisions. Scholarly reviews of the THR debate have highlighted several recent events, including the FDA’s rulings on MRTPs, global THR campaigns launched by multinational tobacco companies, critical responses from World Health Organization (WHO) and tobacco control communities, revisions to the European Union’s Tobacco Products Directive, and the outbreak of e-cigarette or vaping-associated lung injury [3,41].

Although THR has been debated for decades, current conversations increasingly focus on a new generation of tobacco and nicotine products and the regulatory controversies they generate. THR has become a prominent theme in social media discussions related to newer products and their governance [11,42]. However, to date, no published study has systematically analyzed social media posts that explicitly reference “tobacco harm reduction,” classified the valence of these posts (ie, whether they express pro-, anti-, neutral, or mixed perspectives), or compared valence toward THR across countries or regions. It also remains unclear which recent events have driven spikes in THR-related discourse and how these events have shaped global valence.

To address these gaps, this study analyzes THR-related posts on X, examining message valence as well as geographic and temporal trends. By leveraging geo-tagged data, we aim to answer the following research questions (RQs):

1. RQ1: What is the global valence toward THR on X?
2. RQ2: What are the geographic and temporal distributions of valence toward THR?
3. RQ3: What events are associated with increases in THR discourse and shifts in valence?

### Key Themes in Anti- and Pro-THR Posts

In any narrative, including social media posts, authors make deliberate choices in language and structure that frame how an issue is presented. These frames suggest what the issue is fundamentally about and highlight certain keywords or themes that shape how readers understand the topic. Such framing can significantly influence public discourse and the perceived legitimacy or urgency of public health strategies like THR [43].

Previous research has explored how THR narratives have been constructed in news media and industry documents. For instance, 1 study analyzed US news coverage of THR from 1996 to 2014 and found that portrayals of THR shifted over time, reflecting tensions between public health advocates and industry representatives [44]. These tensions centered on the scientific credibility of THR and debates over regulatory issues, such as marketing and taxation. Similarly, another study, drawing on internal tobacco industry documents and interviews, concluded that THR narratives provided strategic benefits to the tobacco industry, renewed their access to policymakers and health organizations, and enhanced reputational standing through corporate social responsibility efforts [45]. Fitzpatrick and colleagues [46] also examined press releases and annual reports from PMI and BAT between 2011 and 2021, identifying dominant themes, such as “capacity and resources,” “health and safety,” and “economic benefits.”

These studies provide valuable insights into recurring themes in THR discourse—particularly those related to science, regulation, and health—and raise the possibility that these themes may have been strategically framed by the industry. However, they are limited in scope, primarily focusing on industry-generated content or US-based mainstream media. These approaches do not fully capture the breadth of stakeholder perspectives emerging on social media, where a wider range of stakeholders participate in shaping global discourse on THR. Although systematic reviews have identified major themes related to e-cigarettes on social media (eg, health effects, cessation support, regulatory debates, and personal testimonials) [47], little research has specifically examined how THR itself is framed on these platforms, particularly in posts that explicitly support or oppose the concept. Thus, this study aims to address the following RQ through thematic analysis:

RQ4: What are the key themes expressed in posts supporting or opposing THR?

### Stakeholder Participation

The authors or accounts behind social media posts about a topic represent a diverse array of stakeholders who actively contribute to and shape relevant discourses. Prior analyses of conversations surrounding newer products have identified key participants, including the tobacco industry, government agencies, tobacco control advocates, public health researchers, media outlets, policymakers, and individuals who use tobacco products. These studies highlight the prominent role played by the industry and its affiliates, ranging from front groups and sponsored influencers to product reviewers, in influencing digital narratives [48-50]. In recent years, additional actors have become increasingly visible in the THR space, including harm reduction–focused foundations and consumer organizations. Some of these groups are directly or indirectly funded by tobacco companies, raising questions about their independence and alignment with industry interests [11].

Given this landscape, this study investigates the presence of industry-affiliated authors, along with THR advocates in THR discourse on social media. Specifically, we examine the proportion of posts authored by tobacco companies, manufacturers, and retailers, as well as the frequency of newer product mentions and marketing attempts. We also analyze the proportion of posts authored by THR advocates, given their growing presence in this discourse. A higher volume of industry-generated content may increase public exposure to proindustry messaging, potentially influencing beliefs and aligning public sentiment with corporate objectives. These insights are critical for understanding how the tobacco industry leverages digital platforms to shape narratives and mobilize online communities, while also situating these efforts alongside independent advocacy voices [7]. Hence, we pose the following RQs:

1. RQ5: Who are the stakeholders participating in THR discourse?
2. RQ6: What is the extent of involvement of the industry and THR advocates in THR discourse, and how does it vary geographically and over time?

## Methods

### Data Collection and Filtering

To collect data on THR discourse on social media, we used the X application programming interface via the analytics platform Netbase Quid Pro (Quid, developed by Netbase Quid).Quid is a commercial data analytics and visualization platform to collect data from X posts discussing THR. Quid aggregates publicly available social media content through licensed third-party firehose access, enabling large-scale collection of posts based on predefined search terms and time frames. Its algorithms use natural language processing and machine learning techniques to classify, cluster, and visualize text data. Quid has been applied in previous analyses of X and other social media platforms, where researchers have relied on data collection as well as its automated classifications of sentiment, author type, and other metadata [51-53]. Quid allows the extraction of original social media posts along with their automated classifications in structured data files. For X posts, these files typically include the original text, post type (original, repost, or reply), URL, sentiment (negative, neutral, positive, or mixed), author handle, follower count, professional or personal descriptors, positive or negative terms, and geographic information at the city, state, and country levels. In our analysis, we relied on Quid’s automated extraction for geography but manually classified all key variables, including the valence toward THR and author type, as described in detail. The initial search encompassed 192 countries where X is available, as supported by Quid. We used 2 sets of search terms: (1) “tobacco” and (2) “harm reduction” OR “reduced/modified harm/risk” OR “alternative” OR “healthier” OR “cleaner.” Our search queries were intentionally designed to capture discourse explicitly referencing tobacco harm reduction as a strategic concept, reflecting its increasing salience in industry communications, advocacy messaging, and policy debates. This approach enabled us to focus on how THR language itself is framed, promoted, and contested in public discourse.

The data collection period spanned from July 2019 to December 2023, covering major events related to THR and the introduction of newer generations of tobacco and nicotine products marketed with THR claims, some of which were authorized as MRTP products by the FDA (eg, General Snus in October 2019). Only English-language posts were included in the analysis.

This query initially yielded 1,67,867 posts. To ensure data quality, we removed irrelevant advertisements, pornographic content, posts generated by automated apps, and duplicates. Researchers then manually screened the remaining content to retain only posts that substantively addressed THR, beyond merely mentioning or hashtagging the term. The final dataset consisted of 17,361 posts, including 9862 original posts and 7499 replies or comments, originating from users in 87 countries.

### Coding Procedure

To examine message valence (RQs 1‐3) and authorship (RQ5), we applied manual coding. Overall, 4 doctoral-level researchers participated in the process. Among them, 2 coded the valence while the other 2 categorized author types. The lead author supervised the coding process. To ensure intercoder reliability, a random subset of 5000 original posts was used for training and iterative refinement. After several rounds of training and adjustment, intercoder reliability reached acceptable levels for all variables (Krippendorff α range=0.90‐1.0). Coders then independently annotated the remainder of the dataset.

### Valence Toward THR

#### Post Valence

The overall attitude expressed in each post toward THR was assessed and categorized into one of four categories: (1) pro-THR, indicating a positive stance (eg, portraying THR as beneficial, appropriate, or effective for individuals, public health, or society); (2) anti-THR, indicating a negative stance (eg, describing THR as risky, harmful, inappropriate, ineffective, or detrimental); (3) mixed, where both pro- and anti-THR valences were present in roughly equal measure, with neither clearly dominant; and (4) none, for posts that were purely informational or factual, lacking any discernible valence for or against THR.

#### Author Types

A total of 4044 unique accounts created THR-related posts. We manually classified each account or “author” based on information provided by Quid, including the author’s X handle, display name, gender, and profile biography on X. Authors were first grouped into 2 broad categories: organizational/official and individual. For organizational accounts, we adapted and modified existing coding frameworks [11,38] to categorize each author into one of 12 initial types. The initial 12 types the country’s tobacco regulation agency (eg, US FDA equivalents); other government agencies besides the FDA; “Big 4” multinational tobacco companies (ie, PMI, BAT, JTI, and Imperial Brands); other tobacco manufacturers or retailers excluding the Big 4; news organizations; harm-reduction advocacy groups, protobacco advocacy groups and media; antitobacco advocacy groups; hospitals or health care organizations; education or research or science institutions; political groups; and public relations or advertising or marketing or promotion or communication agencies. For individual accounts, we assessed and categorized each author’s organizational affiliation using the same 12-type framework where applicable.

The original 12 author types, which included both organizational and individual accounts, were consolidated into seven broader categories for further analysis: (1) the tobacco industry (the Big 4 tobacco companies, as well as other manufacturers and retailers of newer products, and individuals directly affiliated with the industry); (2) the THR advocate (organizations, media outlets, and individuals who explicitly promote THR or advocate for weaker or no regulation of newer products. This category includes both those who may be directly or indirectly affiliated with the industry, as well as independent actors, such as grassroots consumer advocates, academics, and public health care professionals who advocate THR outside of direct industry affiliation); (3) tobacco control advocates (organizations and individuals who support the complete elimination of all forms of tobacco and nicotine and endorse stricter regulation, including entities, such as the WHO and its affiliates); (4) the government (official accounts representing national or regional health institutions or tobacco regulatory agencies); (5) health care providers and scientists (hospitals, health care organizations, research institutions, and individuals affiliated with such organizations); (6) the others (those that could not be clearly classified into the 5 main groups above, such as news media outlets, public relations and marketing agencies, tax-related organizations, and nontobacco-related consumer groups); and (7) the unknown (accounts whose affiliation or identity could not be determined due to insufficient information).

In addition, we coded individual authors for indications of tobacco use (eg, hashtags, such as #iVAPEiVOTE or #vapeenthusiast) and self-reported health expertise (eg, credentials like MD or PhD) to identify tobacco users and self-claimed experts. We relied on the content of their posts and publicly available profile information to inform our author classifications, ensuring compliance with institutional review board guidelines and X’s terms of service throughout the process. In addition, any personally identifiable information was removed or rephrased, including handles and portions of their content for individual authors.

#### Marketing Attempt

To assess the presence of products in each post (RQ6), we conducted quantitative text mining using Voyant Tools (developed by Stéfan Sinclair and Geoffrey Rockwell) to analyze mentions of product names and categories, excluding conventional cigarettes and cigars [54]. We identified four major product categories, along with their common synonyms and representative brand names: (1) e-cigarettes (eg, vapes, vaping, vape pens, e-cigs, e-hookahs, e-cigarettes, ENDS, Vuse, JUUL, and Blu); (2) heated tobacco products, which heat tobacco to release an aerosol containing nicotine (eg, IQOS and HEETS, tobacco sticks, Eclipse, heat-not-burn, and tobacco heating system); (3) smokeless or chewing tobacco (eg, chewing tobacco, snuff, snus, dissolvables, and lozenges); and (4) nicotine pouches (eg, Zyn, On!, and Velo). Each post was coded to indicate whether it mentioned one or more specific products within each category, as well as whether it referenced any product from any of the 4 categories.

In addition, we coded each post for the presence of marketing attempts, defined as the inclusion of either call-to-action phrases or promotional language commonly used in advertising [55]. Posts were considered to contain marketing attempts if they featured call-to-action phrases encouraging immediate user engagement or behavior (eg, “buy now,” “buy today,” “check it out,” “visit [link/website/store],” “order now,” “get yours,” “don’t miss out,” “limited time,” “act now,” “shop now,” “sign up,” “subscribe,” “grab it,” “claim your [offer/product],” and “learn more”) or promotional language that emphasized special offers, exclusivity, or urgency to generate consumer interest or convey value (eg, “free,” “discount,” “sale,” “exclusive,” “offer,” “BOGO” or “buy one get one,” “save [amount/percent],” “best deal,” “limited edition,” “new arrival,” and “must-have”). The presence of either type of language was coded as a marketing attempt.

#### Location

We estimated the country-level origin of 14,121 posts using geographic data provided by Quid. These posts were traced to 87 countries, which were subsequently grouped into 6 continents (Africa, Asia, Europe, North America, South America, and Oceania) and 4 levels of economic development (high, upper-middle, lower-middle, and low), based on the United Nations’ classification [56].

### Statistical Analysis

We conducted a series of statistical analyses to examine differences in THR discourse across time, author type, region, and economic context. Descriptive statistics were first used to summarize distributions of post frequency, author characteristics, and follower counts. To assess group differences in categorical variables (eg, valence by year, author type, geographic region, economic classification, and country), we used chi-square tests of independence. Both nonparametric chi-square tests and Pearson chi-square tests were calculated to confirm the robustness of the results. All analyses were performed using IBM SPSS Statistics 29.0.

### Qualitative Thematic Analysis

To identify key themes in posts (RQ4), we conducted a qualitative inductive thematic analysis of posts expressing either pro-THR or anti-THR valence. We followed the 6-phase process for thematic analysis outlined by Clarke and Braun [57], which includes familiarization with the data, initial coding, development of main themes, refinement of themes, naming of themes, and the final write-up [57,58]. The lead author and a research assistant independently conducted the first 2 phases by reading the posts and assigning initial, concise labels to each post. Posts were allowed multiple labels if applicable. Following this, we collaboratively reanalyzed the dataset, integrated and refined the labels, and codeveloped the final thematic categories.

### Ethical Considerations

This study solely relied on publicly available social media posts on X. To ensure the ethical standard, any personally identifiable information was removed or rephrased, including handles, names of individual authors, and portions of their content. We also used only aggregated measures to protect the identities of individual social media users.

## Results

### Overview

Our final sample consists of 17,361 posts discussing THR that were posted between July 2019 and December 2023. Specifically, 2054 (11.8%) posts were posted in 2019 (starting from July), 3578 (20.6%) in 2020, 3730 (21.5%) in 2021, 2942 (16.9%) in 2022, and 5057 (29.1%) in 2023. Of the 14,121 posts for which country-level location was available, nearly half originated from North America (n=8553, 49.3%), followed by Europe (n=1933, 11.1%) and Asia (n=1924, 11.1%). High-income countries contributed the majority of posts (n=11,739, 67.6%). The United States (n=8090, 46.6%) was the most frequent country of origin, followed by India (n=1484, 8.5%) and the United Kingdom (n=1403, 8.1%).

As for the author, THR advocates contributed the highest proportion of THR posts, accounting for 42.8% (n=7426) of the total, followed by the tobacco industry (n=1042, 6%), health care providers, and scientists (n=1629, 9.4%), tobacco control advocates (n=364, 2.1%), and governments (n=333, 1.9%). A substantial portion of authors (n=5069, 29.2%) were categorized as others. In addition, 21.3% (n=3692) of posts were authored by individuals who were self-identified as tobacco users, and 6.4% (n=1118) of posts were created by individuals claiming to have medical or scientific expertise. In total, there were 4044 unique authors, with an average of 4.29 (SD 26,163) posts per author. The mean follower count per author was 19,009 (SD 2234), while the median was 1042 (IQR 0-19,844,406). Follower counts ranged from as few as 1 to as many as 1,984,406 for the Wall Street Journal (@WSJ) and up to 14,499,829 for the World Health Organization (@WHO). These figures highlight the diverse range of stakeholders participating in THR discussions and underscore the potential reach and influence of these conversations on the platform. Table 1 provides additional details, including the accounts with the highest number of posts, their follower counts, and representative posts for each author type.

**Table 1.** Most active accounts based on author type in THR^a^ discourse and their post counts, example post, and number of followers.

Account handle (description)	Post count, n	Representative post	Followers, n
THR advocates			
User 2^b^	2214	“Tobacco #harmreduction (THR) products were invented because traditional cessation products and 60 years of tobacco control are NOT effective. THR caused the greatest decline in smoking rates ever recorded where they’re”	1230
User 3^b^	335	“Tobacco harm reduction opponents just can’t accept tobacco companies now seriously threatened by #Vaping etc though market capitalisation almost halved 4 years! “Phillip Morris International has gone as far as to argue that it has a ‘smoke-free future’”	9762
User 4^b^	288	“Smoking harms devastate marginalized populations, low-income, minorities, mental health diagnosis, incarcerated, LGTBQ+ & homeless people. Tobacco Harm Reduction is about health equity.”	1607
@PVapes (Planet of the Vapes; UK vaping community site)	156	“The EU Commissions’ misguided attacks on harm reduction continue, says a campaign organization, as the EU moves to ban all flavored heated tobacco products”	27,242
@thr101org (Tobacco Harm Reduction 101; THR news media)	151	“#DidYouKnow - A 2019 study found e-cigarettes to be 2x effective than traditional nicotine replacement therapy (gum, patch, lozenges) in helping smokers quit???”	721
Tobacco industry			
@SmokeFreeFdn (Smoke Free Foundation by PMI^c^)	53	“Today is #internationalharmreductionday2021. Although there will be 8 million deaths due to smoking this year, we are still lacking a sense of urgency for #tobaccoharmreduction solutions. #IHRD21”	3851
@RedDevil680 (Vape and CBD Shop	48	“@DoctorXXX Doctor. I have seen some of your posts against vaping. How can you logically accept harm reduction in accidents with the use of seat belts and airbags yet not the same thing for vape as harm reduction for tobacco use? Do you see The logical disconnect here?”	846
@AltriaScience (Altria Science)	45	“We invest in the core science needed to demonstrate that smoke-free products are less harmful than cigarettes. We’ve recruited scientists from around the world, from many different disciplines, who share a common goal of tobacco harm reduction*.”*	228
@EsonCorporation^b^ (owner of the brand NEAFS; tobacco free sticks)	45	“NEAFS strawberry heated sticks, Tobacco free heated alternative. Online sales available now for UK only (additional EU countries available in September). Get NEAFS now! #NEAFS #TEO #smokefree #tobaccofree #nicotine”	9
User 1^b^	44	“@WHO.18 shareholder states of tobacco companies that are members of the WHO Anti-Tobacco Convention (FCTC): a conflict of interest? 7 protect cigarette sales by prohibiting their population from reducing risks with vaping.”	2058
Health care providers			
User 5^b^ (developmental neurobiologist, PhD)	497	“Studies like that just demonstrate how much #JunkScience is now being produced by #HarmReduction-denialist researchers in old-school tobacco control. Bias is created by the Lysenkoist-like nature of that field today.”	10,168
User 6^b^ (public health scientist)	23	“Good summary of Big Tobacco’s ecig Trojan Horse which has legitimized their existence and influence while addicting a new generation. “Harm Reduction” and their promoters are simply part of the industry’s plan to weaken or delay tobacco control policies.”	353
@CalNcpc (Nicotine and Cannabis Policy Center in University of California Merced)	11	“Do you still believe #SmokelessTobaccoProducts like #ChewingTobacco are a #SaferAlternative? Well, according to @MayoClinic, these products pose similar #HealthRisks like #addiction, #HeartDisease, #DentalDisease & more! Don’t be fooled, #QuitTobacco today”	178
Tobacco control advocates			
@TobaccoFreeKids (Campaign for Tobacco-Free Kids)	10	“Tobacco companies’ claims about “harm reduction” are really a cynical smokescreen designed to divert attention from their true goals: to maximize profits by perpetuating addiction. Don’t fall for the act. #TakeDownTobacco”	27,484
@IndiaVsTobacco (Envisioning an India that is healthier, happier, and tobacco-free)	9	“Some lessons aren’t on the syllabus. Let’s keep tobacco out of our schools’ radius. Say NO to easy accessibility of tobacco to our youth. Together, we’re scripting a healthier future at promisetoprotect.co.in! #ProtectOurYouth #TobaccoFreeIndia #IndiaQuitsTobacco”	1519
@QuitandStayQuit (Guidebook for nicotine cessation)	8	“Stressed? Bored? Angry? Recognize your moods and find healthier ways to feel better without tobacco. #QuitTobacco #QuitVaping”	718
Governments			
@FDATobacco (US FDA Center for Tobacco Products)	27	“Today…FDA posted more materials from 22nd Century Group’s modified risk tobacco product (MRTP) applications for VLN(TM) King and VLN(TM) Menthol King “very low nicotine” combusted, filtered cigarettes.”	43,946
@Healthy_MoCo (Montgomery County)	13	“Learn strategies for managing stress and how to quit tobacco by joining free weekly sessions by the Freedom From Smoking Group Clinic…Is Vaping Better Than Smoking? If you think vaping is a healthier, safer or ““better”” alternative to smoking or you’re using e-cigarettes to try to quit smoking, think again. Vaping has many dangers and is considered a public health concern.”	703
@PlumasHealth (Quincy Public Health Agency)	11	“Nicotine, butane, ethylphenol are just a few of the dangerous chemicals found in cigarette butts. It’s time for Big Tobacco to come clean about the dangers of their toxic waste.”	135
@HealthyBoston (Boston Public Health Commission)	9	“Vaping is NOT a healthy alternative to tobacco. Do not be misled. “E-juice” contains harmful chemicals, heavy metals and other substances that are addictive and linked to fatal diseases. Do not start. If you need help quitting:”	26,094
@Scotthealthdept (Scott County Health Department)	9	“Smokeless tobacco is associated with numerous health problems. According to the CDC, smokeless tobacco can cause white or gray patches to form inside the mouth that can lead to cancer. It is not a safe alternative to smoking.”	222
Others/Unknown			
User 7^b^	138	“All tobacco harm reduction deniers are hypocrites banging on everyone to get vaccinated yet lobby & demonize safer nicotine alternatives. Shows complete nonsense of their arguments. Vaping is vaccine of tobacco harm. Science says #Vaping wont kill. Science says vaccines can kill.”	2017
User 8^b^	73	“Taking away an effective tool of tobacco harm reduction from those who fight for and protect our freedom of choice is outrageous in addition to the fact that many became addicted to government issued cigarettes first”	186
@Protectaxpayers (Taxpayers Protection Alliance)	36	“As states continue to demonize tobacco harm reduction products and makers, it is apparent tobacco and vape companies are doing the better job at reducing smoking rates among youth and young adults”	4595
@RegWatchCanada (Regulator Watch news media)	34	“Doubling The Tax On Tobacco-Free Alternatives Leaves Smokers With No Choice But To Smoke On”	1997
User 9^b^	32	“why tobacco harm reduction makes good business sense…”	23,766

a THR: tobacco harm reduction.

b Any personally identifiable information was removed or rephrased, including handles, names of individual authors, and portions of their content.

c PMI: Philip Morris International.

### Valence Toward THR

Table 2 presents the distribution of valence toward THR by year, author type, and geographic region. Overall, 71.4% (12393/17,361) of posts expressed a pro-THR valence, significantly more frequent than anti-THR (3925/17,361, 22.6%), mixed (63/17,361, 0.4%), or none (980, 5.6%), with statistical significance confirmed by nonparametric chi-square tests (*χ*²_3_=21,797.2; *P*<.001).

**Table 2.** Valence toward Tobacco harm reduction by year, author, and geographic location (N=17,361).

Variable	Posts, n (%)	Pro-THR^a^, n (%)	Anti-THR, n (%)	Mixed, n (%)	None, n (%)	Nonparametric chi-square test (*df*)	Pearson chi-square (*df)*
Total		12,393 (71.4)	3925 (22.6)	63 (0.4)	980 (5.6)	21797.2^c^ (3)	—^b^
Year							285.1 (12)^c^
2019	2054 (11.8)	1549 (75.4)	380 (18.5)	15 (0.7)	110 (5.3)	2924.9^c^ (3)	
2020	3578 (20.6)	2599 (72.6)	698 (19.5)	5 (0.1)	276 (7.7)	4603.3^c^ (3)	
2021	3730 (21.5)	2497 (66.9)	963 (25.8)	16 (0.4)	254 (6.8)	4022.8^c^ (3)	
2022	2942 (16.9)	1902 (64.6)	815 (27.7)	19 (0.6)	206 (7)	2938.3^c^ (3)	
2023	5057 (29.1)	3848 (76.1)	1067 (21.1)	10 (0.2)	132 (2.6)	7557.6^c^ (3)	
Author							
Tobacco industry	1042 (6)	973 (93.3)	26 (2.5)	0 (0)	43 (4.1)	1409.7^c^ (3)	5,371.9 (24)^c^
THR advocates	7426 (42.8)	7084 (95.4)	104 (1.4)	15 (0.2)	223 (3)	19,660.3^c^ (3)	5,371.9 (24)^c^
Tobacco control advocates	364 (2.1)	84 (23.1)	256 (70.3)	3 (0.8)	21 (5.8)	466.9^c^ (3)	5,371.9 (24)^c^
Governments	333 (1.9)	24 (7.2)	276 (82.9)	0 (0)	33 (9.9)	368.2^c^ (3)	5,371.9 (24)^c^
Health care providers	1629 (9.4)	716 (44)	826 (50.7)	10 (0.6)	77 (4.7)	1319.9^c^ (3)	5,371.9 (24)^c^
Others	5069 (29.2)	2813 (55.5)	1779 (35.1)	36 (0.7)	441 (8.7)	3829.8^c^ (3)	5,371.9 (24)^c^
Unknown	1498 (8.6)	776 (51.8)	586 (39.2)	1 (0.1)	135 (9)	1199.6^c^ (3)	5,371.9 (24)^c^
Tobacco users	3692 (21.3)	3618 (98)	15 (0.4)	0 (0)	59 (1.6)	6941.6^c^ (3)	—
Self-claimed experts	1118 (6.4)	934 (83.5)	143 (12.8)	7 (0.6)	34 (3)	2080.6^c^ (3)	—
Continent^d^							2,497.7 (15)^c^
Africa	468 (2.7)	294 (62.8)	122 (26.1)	7 (1.5)	45 (9.6)	415.7^c^ (3)	
Asia	1924 (11.1)	592 (30.8)	614 (31.9)	4 (0.2)	714 (37.1)	2070.9^c^ (3)	
Europe	1933 (11.1)	1625 (84.1)	199 (10.3)	12 (0.6)	97 (5)	3633.4^c^ (3)	
North America	8553 (49.3)	6491 (75.9)	1540 (18)	26 (0.3)	496 (5.8)	12358.5^c^ (3)	
South America	10 (0.1)	3 (30)	7 (70)	0 (0.)	0 (0.)	1.6^e^ (3)	
Oceania	1234 (7.1)	1084 (87.8)	98 (7.9)	4 (0.3)	48 (3.9)	2613.5^c^ (3)	
Economy^d^							2,135.9 (9)^c^
High income	11739 (67.6)	9193 (78.3)	1855 (15.8)	35 (0.3)	656 (5.6)	18381.0^c^ (3)	
Upper-middle income	2062 (11.9)	1242 (60.2)	677 (32.8)	17 (0.8)	126 (6.1)	218.9^c^ (3)	
Lower-middle income	244 (1.4)	82 (33.5)	149 (61.2)	1 (0.3)	12 (5)	1972.1^c^ (3)	
Low income	72 (0.4)	57 (79.2)	13 (18.1)	2 (2.8)	0 (0)	70.5^c^ (3)	
Top 11 countries^d^							—
United States	8090 (46.6)	6156 (76.1)	1456 (18)	25 (0.3)	453 (5.6)	11806.3^c^ (3)	
India	1484 (8.5)	382 (25.6)	1052 (70.6)	3 (0.2)	47 (3.6)	1873.2^c^ (3)	
United Kingdom	1403 (8.1)	1228 (87.5)	106 (7.6)	10 (0.7)	59 (4.2)	2932.1^c^ (3)	
Australia	1139 (6.6)	1001 (87.9)	87 (7.7)	5 (0.4)	46 (4)	2414.5^c^ (3)	
Canada	439 (2.5)	312 (71.1)	80 (18.2)	0 (0)	47 (10.7)	285.0^c^ (3)	
Pakistan	207 (1.2)	101 (48.8)	98 (47.3)	0 (0)	8 (3.9)	80.9^c^ (3)	
Nigeria	139 (0.8)	86 (61.9)	40 (28.8)	0 (0)	13 (9.4)	58.8^c^ (3)	
South Africa	121 (0.7)	87 (71.9)	21 (17.4)	2 (1.7)	11 (9.1)	147.9^c^ (3)	
Germany	115 (0.7)	94 (81.7)	16 (13.9)	0 (0)	5 (4.3)	122.8^c^ (3)	
Kenya	111 (0.6)	51 (45.9)	42 (37.8)	3 (2.7)	15 (13.5)	54.7^c^ (3)	
New Zealand	95 (0.5)	83 (87.4)	10 (10.5)	0 (0)	2 (2.1)	125.8^c^ (3)	

a THR: Tobacco harm reduction.

b Not available.

c *P*<.001

d Region, income, and top 11 countries were based on 14,121 posts for which location data was available.

e *P*=.20

Pro-THR sentiment was dominant across most author types. THR advocates contributed the highest proportion of pro-THR posts (7084/7426, 95.4%), followed by tobacco users (3618/3692, 98.0%), the tobacco industry (973/1042, 93.3%), and self-claimed experts (934/1118, 83.5%). In contrast, anti-THR valence was most prominent among government accounts (276/333, 82.9%), tobacco control advocates (256/364, 70.3%), and health care providers (826/1629, 50.7%). Posts by authors classified as “others” (5069/17,361, 29.2%) and “unknown” (1498/17,361, 8.6%) showed a more mixed distribution, with 55.5% (2813/5069) and 51.8% (776/1498) pro-THR, and relatively higher shares of anti-THR posts (1779/5069, 35.1% and 586/1498, 39.2%), respectively. Health care providers exhibited a nearly even split between pro-THR (716/1629, 44%) and anti-THR (826/1629, 50.7%) valence, while tobacco control advocates and government accounts were overwhelmingly anti-THR. These differences in THR valence across author types, including tobacco users and self-claimed experts, were statistically significant, as confirmed by both nonparametric and Pearson chi-square tests.

Regionally, pro-THR valence was most prevalent in Oceania (1084/1234, 87.8%), followed by Europe (1625/1933, 84.1%), North America (6491/8553, 75.9%), Africa (294/468, 62.8%), and Asia (592/1924, 30.8%). Anti-THR valence was most notable in Asia (614/1924, 31.9%) and Africa (122/468, 26.1%), while South America, though based on a small sample (n=10), showed a predominance of anti-THR sentiment (7/10, 70%). Oceania and Europe had the highest shares of pro-THR posts and the lowest levels of anti-THR valence.

Across economic development groups, low-income countries showed the highest proportion of pro-THR posts (57/72, 79.2%), followed by high-income countries (9193/11739, 78.3%), upper-middle-income (1242/2062, 60.2%), and lower-middle-income countries (82/244, 33.5%). Anti-THR valence was most prominent in lower-middle-income countries (149/244, 61.2%).

Among the top 11 countries by volume of THR-related posts, Australia (1001/1139, 87.9%), the United Kingdom (1228/1403, 87.5%), and New Zealand (83/95, 87.4%) exhibited the highest proportions of pro-THR valence. The United States also showed a strong pro-THR leaning (6156/8090, 76.1%). In contrast, India (1052/1484, 70.6%), Pakistan (98/207, 47.3%), and Kenya (42/111, 37.8%) displayed the highest levels of anti-THR valence. Notably, Kenya also had the highest proportion of neutral or non-valence posts (15/111, 13.5%). These patterns indicate substantial variation in THR attitudes by geography, economic context, and author affiliation, with statistically significant differences observed across all comparisons (*P*<.001).

### Peaks of THR Discourse and Associated Events

**Figure 1.**
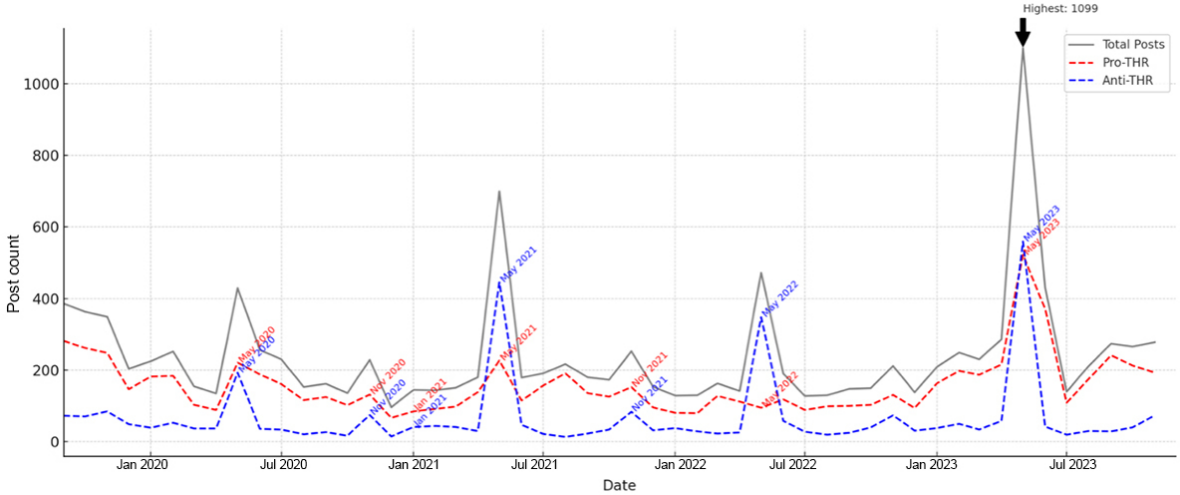
Monthly trends in tobacco harm reduction (THR) discourse and pro- and anti-THR valence (July 2019-December 2023).

Figure 1 displays the monthly trends in the volume and valence of THR posts between July 2019 and December 2023. Six prominent peaks in post volume were identified, each corresponding to key regulatory or advocacy events. Spike months (those with >100% increase from the previous month) are annotated with dates. Table 3 provides an overview of these peaks, including the number of pro- and anti-THR posts within each month, the percentage increase compared to the previous month, and representative pro- and anti-THR narratives during those periods.

**Table 3.** Key events and anti- and pro-THR^a^ narratives during peaks in THR discourse.

Month and year	Total posts, n (% Δ)	Key event	Pro-THR, n (% Δ)	Examples	Anti-THR, n (% Δ)	Examples
September 2019	386	E-cigarette and Vaping Associated Lung Industry (EVALI) outbreak and calls for flavored vape bans	282	“What ’vaping epidemic’? Vaping helped me stop smoking after 30 y. That’s not an epidemic.”	73	“I don’t understand how ppl can say I didn’t know nicotine was in the vape. Do y’all not read? It’s not a secret”
November 2019	349 (–3.9)	US FDA^b^ authorizes General Snus under MRTP^c^	248 (–5.3)	“Using General Snus instead of cigarettes puts you at a lower risk of mouth cancer, heart disease. #TobaccoHarmReduction.”	85 (+19.7)	“You’re promoting snus as a safer alternative? Are you fxxxx retarded?”
May 2020	429 (+22.9)	EU^d^ menthol cigarette ban and NY^e^ State flavored vape ban	221 (–10.9)	“Smokers might respond better to a menthol ban if they had clear alternatives.”	193 (+127.1)	“Menthol marketing misleads young people. FDA should act like MA and prevent further disease.”
May 2021	699 (+62.9)	WHO’s^f^ World No Tobacco Day: “Commit to Quit”	227 (+2.7)	“WHO should embrace innovation and include THR in its strategy. Learn about #HarmReduction.”	445 (+130.6)	“ERS supports WHO FCTC but does not recommend THR as a population-based strategy.”
May 2022	472 (+32.5)	FDA orders JUUL to halt sales	95 (+58.1)	“Flavor bans have no effect. Will anti-vaping zealots ever admit THR works?”	348 (+21.8)	“JUUL & Zyn are just diet versions of Big Tobacco.”
May 2023	1099 (+284.3)	WHO’s World No Tobacco Day: “Grow food, not tobacco”	522 (+141.7)	“Drop political agendas & embrace #HarmReduction. Smokers’ lives matter more than your [WHO] funding.”	559 (+863.8)	“Public health officials allow harmful products like ecigs for kids who never smoked. #NotHarmReduction”
September 2023	274 (+29.2)	Cancer awareness month	241 (+36.9)	“Countries like Sweden, England, and New Zealand are showing success in reducing cancer through tobacco harm reduction—why aren’t others following?”	29 (–3.3)	“Beat the odds against cancer! Here’s why quitting all tobacco—including vaping—is the safest path.”

a THR: tobacco harm reduction.

b FDA: US Food and Drug Administration.

c MRTP: modified risk tobacco product.

d EU: European Union.

e NY: New York.

f WHO: World Health Organization.

**Figure 2.**
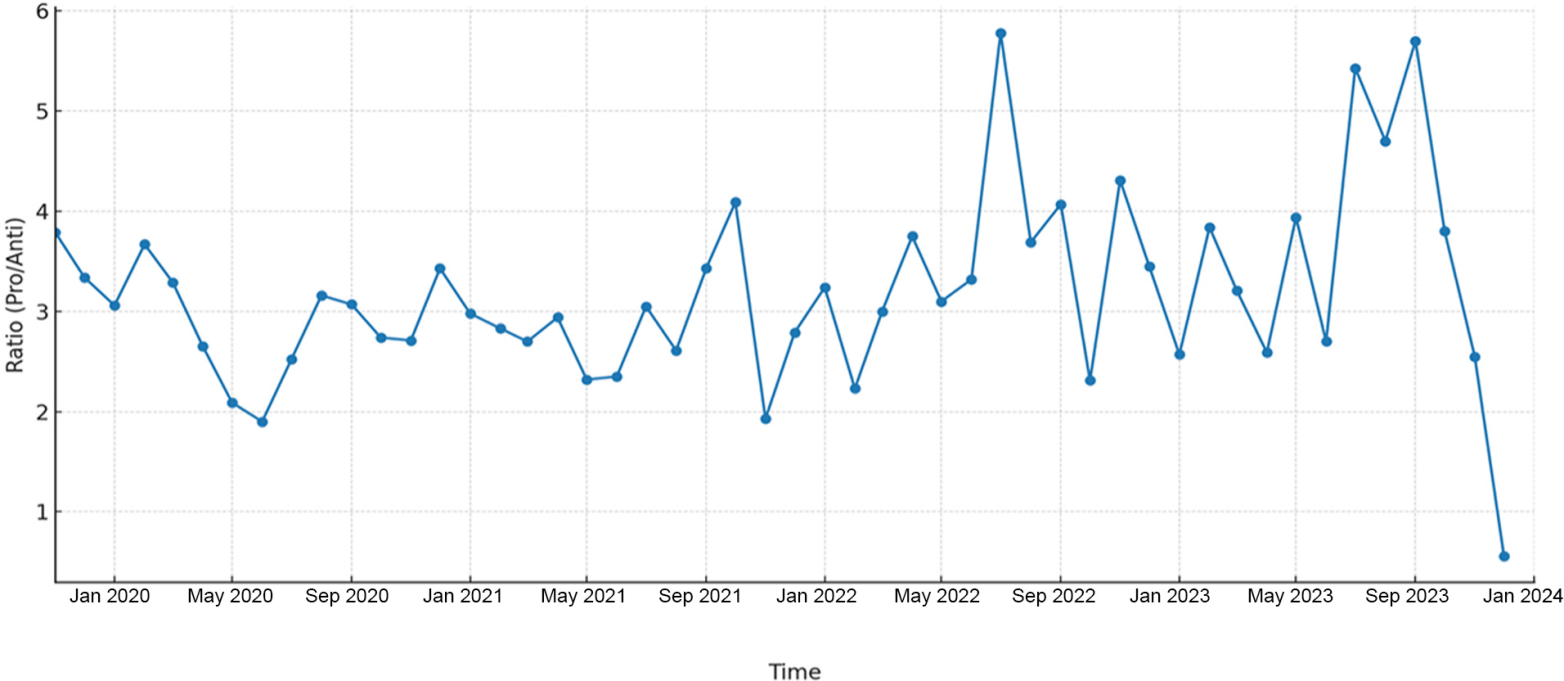
Pro- to anti-THR ratio of tobacco harm reduction discourse over time (July 2019-December 2023). THR: tobacco harm reduction.

Figure 2 also shows the pro-THR to anti-THR ratio over time between July 2019 and December 2023. Ratios consistently above 1.0 indicate that pro-THR voices outnumbered anti-THR voices throughout most of the period. Peaks in mid-2022, mid-2023, and September 2023 reflect moments of particularly strong pro-THR dominance. The trend, however, also reveals fluctuations, with lower ratios in mid-2020, late-2021, and mid-2023. A sharp decline in December 2023 marks the only point where anti-THR voices overtook pro-THR voices in the dataset.

Ratios above 1.0 indicate that pro-THR posts outnumbered anti-THR, as shown in Figure 2. The first notable peak occurred in November 2019 (n=349), following the FDA’s first-ever decision to authorize the marketing of Swedish Match’s General Snus as an MRTP on October 22, 2019 [59]. Anti-THR valence increased (n=85, +19.7%), with more posts condemning the promotion of snus as a safer alternative to smoking, while pro-THR valence remained the majority (n=248, –5.3%).

The second peak emerged in May 2020 (n=429, +22.9%), coinciding with the implementation of the European Union’s ban on menthol cigarettes [60] and New York State’s ban on flavored nicotine vapor products [61]. Both pro-THR (n=221, –10.9%) and anti-THR (n=193, +127.1%) narratives responded strongly. Pro-THR posts advocated for alternative options for menthol smokers, while anti-THR posts argued that flavored products have been used to target youth.

A larger peak appeared in May 2021 (n=699, +62.9%), driven by global discourse surrounding the WHO’s World No Tobacco Day campaign titled “Commit to Quit” [62]. Anti-THR valence rose sharply (n=445, +130.6%), and pro-THR posts increased modestly (n=227, +2.7%).

In May 2022, discourse spiked again (n=472, +32.5%) following major events, such as World No Tobacco Day and the FDA’s issuance of marketing denial orders for JUUL Labs Inc, which mandated a halt to the company’s sales and distribution [63]. Pro-THR posts (n=95, +58.1%) surged, with many emphasizing the harm reduction potential of JUUL products. Anti-THR valence also increased (n=348, +21.8%), including critiques of JUUL and broader tobacco industry tactics.

The most substantial spike occurred in May 2023 (n=1099, +284.3%), once again linked to World No Tobacco Day, this time themed “Grow food, not tobacco” [64]. Both anti-THR (n=559, +863.8%) and pro-THR (n=522, +141.7%) valence intensified. Pro-THR posts advocated THR as a viable public health strategy while anti-THR messages criticized the continued access to newer products, particularly among youth.

The final observed peak occurred in September 2023 (n=274, +29.2%), coinciding with broader discourse tied to Cancer Awareness Month. Pro-THR valence increased by 36.9% (n=241), with many highlighting THR as a cancer prevention strategy. In contrast, anti-THR valence (n=29, −3.3%) continued to advocate for the cessation of all tobacco and nicotine products in the context of cancer prevention.

### Industry and THR Advocate Participation

Table 4 presents the distribution of participation in THR-related posts by industry and THR advocates, disaggregated by year, region, economy, and country. Posts authored by the Big 4 accounted for 1.1% (193/17,361) of the dataset. The most active accounts included @SmokeFreeFdn (PMI’s Smoke-Free Foundation; n=53, 27.5%), @AltriaScience (PMI’s parent company; n=45, 23.3%), @InsidePMI (n=27, 14%), @BAT_Sci (n=20, 10.4%), and @BATplc (n=17, 8.8%). Other contributors included @BE_BAT_official, @EUOffice_BAT, @ImperialBrands, @PhilipMorrisMY, @philipmorrisza, @ploom_uk (JTI’s heated tobacco brand), and @PMIScience. Participation by the Big 4 peaked in 2022 (63/2942, 2.1%) and was relatively stable in other years, ranging from 0.3% (6/2054) in 2019 to 1.1% (55/5057) in both 2020 and 2023. Although limited in volume, Big 4 accounts played a visible role in shaping THR discourse. Posts from other tobacco manufacturers and retailers contributed 4.9% (849/17,361) of the dataset, peaking at 6.8% (199/2942) in 2022. THR advocates contributed to 42.8% (7426/17,361) of posts, with steadily increasing participation from 28% (575/2054) in 2019 to 59.6% (3014/5057) in 2023.

**Table 4.** Participation of industry and THR^a^ advocates by year, geographic location, and income level.

	Account type			Post content	
	By industry		By THR advocates	Newer product appearance	Marketing attempt
	By Big 4	By makers or retailers			
Total, n (%)	193 (1.1)	849 (4.9)	7426 (42.8)	6881 (39.6)	2724 (15.7)
Time, n (%)					
2019 (n=2054)	6 (0.3)	95 (4.6)	575 (28)	1169 (56.9)	211 (10.3)
2020 (n=3578)	38 (1.1)	193 (5.4)	1325 (17)	1653 (46.2)	439 (12.3)
2021 (n=3730)	31 (0.8)	126 (3.4)	1381 (37)	1310 (35.1)	445 (11.9)
2022 (n=2942)	63 (2.1)	199 (6.8)	1131 (38.4)	1018 (34.6)	419 (14.2)
2023 (n=5057)	55 (1.1)	236 (4.7)	3014 (59.6)	1731 (34.2)	1210 (23.9)
Pearson chi-square (*df*)	43.7^b^ (4)	43.3^b^ (4)	889.3^b^ (4)	445.3^b^ (4)	381.1^b^ (4)
Region, n (%)					
Africa (n=468)	0 (0)	5 (1.1)	169 (36.1)	90 (19.2)	48 (10.3)
Asia (n=1924)	2 (0.1)	4 (0.2)	300 (15.6)	253 (13.1)	339 (17.6)
Oceania (n=1234)	0 (0)	0 (0)	822 (66.7)	587 (47.6)	87 (7.1)
Europe (n=1933)	67 (3.5)	180 (21.3)	1104 (57.1)	1048 (54.3)	239 (12.4)
North America (n=8553)	79 (0.9)	462 (54.4)	3473 (40.6)	3732 (43.6)	1468 (17.2)
South America (n=10)	0 (0)	0 (0)	0 (0)	5 (50)	2 (20)
Pearson chi-square (*df*)	139.6^b^ (6)	265.9^b^ (6)	1099.9^b^ (6)	925.9^b^ (6)	118.2^b^ (6)
Economy, n (%)					
High (n=11,739)	146 (1.2)	634 (5.4)	5396 (46)	5370 (45.7)	1805 (15.4)
Upper middle (n=2062)	2 (0.8)	17 (7)	372 (18)	257 (12.5)	333 (16.1)
Lower middle (n=244)	0 (0)	1 (0.4)	60 (24.6)	80 (32.8)	46 (18.9)
Low (n=72)	0 (0)	0 (0)	40 (55.6)	8 (11.1)	0 (0)
Pearson chi-square (*df*)	28.2^b^ (3)	126.2^b^ (3)	638.5^b^ (3)	867.1^b^ (3)	18.6^b^ (3)
Top 11 countries, n (%)					
United States (n=8090)	79 (1.0)	428 (5.3)	3344 (41.3)	3486 (43.1)	1414 (17.4)
India (n=1484)	0 (0)	1 (0.1)	255 (17.2)	169 (11.4)	261 (17.6)
United Kingdom (n=1403)	52 (3.7)	148 (10.5)	902 (64.3)	812 (57.9)	181 (12.9)
Australia (n=1139)	0 (0)	0 (0)	768 (67.4)	522 (45.8)	76 (6.7)
Canada (n=439)	0 (0)	25 (5.7)	126 (28.7)	233 (53.1)	59 (13.4)
Pakistan (n=207)	0 (0)	0 (0)	18 (8.7)	14 (6.8)	34 (16.4)
Nigeria (n=139)	0 (0)	0 (0)	38 (27.3)	28 (20.1)	9 (6.5)
South Africa (n=121)	0 (0)	5 (4.1)	53 (43.8)	43 (35.5)	17 (14)
Germany (n=115)	0 (0)	7 (6.1)	53 (46.1)	68 (59.1)	10 (8.7)
Kenya (n=111)	0 (0)	0 (0)	41 (36.9)	9 (8.1)	19 (17.1)
New Zealand (n=95)	0 (0)	0 (0)	54 (56.8)	65 (68.4)	11 (11.6)

a THR: tobacco harm reduction.

b *P*<.001

Posts mentioning newer products appeared in 39.6% (6881/17,361) of the dataset, most frequently in 2019 (1169/2054, 56.9%) and declining to 34.2% (1731/5057) by 2023. Marketing attempts were identified in 15.7% (2724/17,361) of posts, increasing notably from 10.3% (211/2054) in 2019 to 23.9% (1210/5057) in 2023, reflecting a shift toward more promotional communication. These year-over-year changes were statistically significant (*P*<.001).

Regionally, North America accounted for more than half of the industry posts (462/8553, 54.4%) and product mentions (3732/8553, 43.6%), while Europe had the highest proportion of Big 4 content (67/1933, 3.5%) and product appearance (1048/1933, 54.3%). Oceania and Europe had the strongest THR advocate presence (822/1234, 66.7% and 1104/1933, 57.1%, respectively). Asia stood out for its relatively higher share of marketing attempts (339/1924, 17.6%). All regional variations in participation and content were statistically significant (*P*<.001).

By economic classification, high-income countries accounted for the highest levels of both Big 4 (146/11,739, 1.2%) and other industry (634/11,739, 5.4%) participation, as well as THR advocacy (5396/11,739, 46%). Upper-middle-income countries showed moderate engagement by the industry (17/2062, 7%) and THR advocates (372/2062, 18%), while low-income countries—though representing a small share of the sample—had the highest proportion of THR advocates (40/72, 55.6%) and no Big 4 participation. Lower-middle-income countries, by contrast, had the highest rates of product mentions (80/244, 32.8%) and marketing attempts (46/244, 18.9%) despite low overall activity. The presence of THR advocates (*χ*²_4_=638.5), product (*χ*²_3_=867.1), and marketing attempt (*χ*²_3_=18.6) all differed significantly across income levels.

Among the top 11 countries, the United States posted the highest levels of tobacco industry participation (428/8090, 5.3%) and Big 4 presence (79/8090, 1%). The United Kingdom, Australia, and New Zealand demonstrated high levels of THR advocacy (64.3%‐67.4%) and frequent product mentions but little to no industry involvement. Pakistan and Nigeria, despite lacking direct Big 4 or industry participation, had relatively high rates of product appearance and marketing attempts.

### Themes in Pro- and Anti-THR Narratives

Prominent themes emerged from both pro- and anti-THR valence groups during the thematic analysis (RQ4).

#### Pro-THR Narratives

##### Overall

Among pro-THR posts, 8 distinct themes emerged. A total of 6885 posts were assigned a single theme, 1701 had 2 themes, and 9 posts were coded with more than 3 themes. Frequently used hashtags included: #HarmReduction (n=2151), #TobaccoHarmReduction (n=1392), #Vaping (n=1091), #VapingSavesLives (n=609), #THR (n=562), #Vape (n=515), #CancerMoonshot (n=506), #WeVapeWeVote (n=456), #Tobacco (n=439), and #SaferNicotine (n=270). Table 5 presents each theme and inclusion and exclusion criteria alongside illustrative excerpts.

**Table 5.** Pro-THR^a^ narratives: Key themes, inclusion and exclusion criteria, and examples.^b^

Theme	Posts, n (%)	Inclusion and exclusion criteria	Representative examples
Advocacy for Safer Alternatives	7602 (61.3)	Posts that make clear comparative claims about the safety or risk-reduction benefits of newer products; it excludes posts that merely describe product use without reference to harm reduction or that focus only on personal experience, which are classified under *Personal Testimonials*.	”Vaping is 95% safer than smoking. It’s called tobacco harm reduction and our president has made a great choice to not ban life saving devices.” ”You’re just fear mongering, which will keep people smoking deadly tobacco cigarettes. Vaping is harm reduction for people who smoke.” ”Stop Smoking, Start Vaping’ is a great evidence-based resource for smokers and doctors.”
Global Models and Comparative Policy Successes	3201 (25.8)	Posts that reference specific countries or regions and make comparative statements about THR policies or outcomes, while excluding general endorsements of THR without geographic or policy comparisons.	“Sweden has perfect proof of concept for tobacco harm reduction. But if its tobacco control activists only focused on ’harm,' there would soon be nothing for them to do.” ”Sweden is about to be the first smoke-free country, and their cancer rates are the lowest in the EU. They embraced THR.” ”Australia’s tobacco control policies -- including extreme hostility toward #SaferNicotine alternatives -- have achieved this A complete fail. It hasn’t worked. So now the government is doubling-down on policies that obviously don’t work.” “Countries like Sweden, England, New Zealand, and Japan have reduced cancer rates by embracing tobacco harm reduction products all with very little youth use. Time is now to save adults who can’t quit smoking from cancer, COPD, heart disease and death.”
Distrust of Public Health Institutions and Financial Influence	1012 (8.2)	Posts that explicitly mention institutions, agencies, or funders and cast doubt on their credibility, motives, or financial or political interests, and it excludes more general critiques of THR opposition that do not identify a specific institution or funding influence.	“@WHO @[DrPublicHealth] are for sale to the highest funder. They will now do their best to “whitewash” reputations & push agendas for $ like denying tobacco #harmreduction that has saved millions of smokers lives.” “@MikeBloomberg is spending $150 million per year on tobacco control to fund NGOs that are required to fight AGAINST tobacco #HarmReduction. Every @BloombergDotOrg tobacco control grantee is, thus, OBVIOUSLY biased and has a massive conflict of interest.” “Although @FDA has banned 99.9% of vaping products and rejected tobacco harm reduction, it’s willing to take a sensible harm-reduction approach with other products. Why the double standard? The agency is run by hypocrites and motivated by politics.” ”@FDATobacco @CDC…Are US agencies genuinely committed in encouraging people away from smoking?”
Endorsement of Industry Efforts for THR	158 (1.3)	Posts that defend adult rights to access safer products or argue that youth-centered narratives and regulations unfairly undermine adult autonomy, while excluding posts that mention youth or adult use without linking the argument to autonomy, regulation, or rights.	”There is a human right to truth-telling, informed consent, freedom of choice, and #HarmReduction… BAT is right: Adult access to #SaferNicotine alternatives to deadly cigarettes is a #HumanRight.” ”@FCT @WHO [@DrXXs] Instead of being praised for providing safer alternatives, the tobacco industry is demonized. They’re doing more to reduce harm than the WHO.” ”As smoking has become a worldwide epidemic of addiction and disease, the companies who caused it now ironically hold the solution. Heated tobacco, vapes, and pouches are changing the game.” ”I don’t trust tobacco companies, but suspect PMI is shifting as fast as it can from deadly products to #SaferNicotine alternatives. And YourOrg [tobacco control advocacy group] is paying tens of millions to prevent that.”
Defense of Adult Autonomy and Critique of Youth-Centered Regulation	304 (2.5)	Posts that defend adult rights to access safer products or argue that youth-centered narratives and regulations unfairly undermine adult autonomy, while excluding posts that mention youth or adult use without linking the argument to autonomy, regulation, or rights.	”@AmericaHeartVA ….Youth vaping is a non-issue, but the greedy non-profits and governments want you to think it is.” ”@CDCTobaccoFree Now we know youth uptake of nic vaping is not a serious issue the priority must shift to saving adults who smoke from cancer, COPD, heart disease, and death” ”Nobody is suggesting kids vape. But adults shouldn’t be denied life-saving options because of the bad decisions of a few teens.”
Criticism of Anti-THR Narratives as Misinformation	379 (3.1)	Posts that explicitly attack or discredit anti-THR actors, narratives, or campaigns as deceptive, misleading, or harmful, and it excludes pro-THR advocacy that does not directly reference or criticize anti-THR actors.	“@ParentsvsVape Lies. Fake News.You are a healthier world’s worst enemy. Dehumanizing us may make it easier for you to sleep at night. In reality, you must own that for millions of adults, vaping flavored nicotine e-liquids has been the only Tobacco Harm Reduction method that worked for them.” ”@[ProfTobaccoControl], You claim vaping equals smoking. That’s false. Millions of lives are being saved by vaping, it’s time you acknowledge it. You’re a racist sexual predator, stick to improving your behavior and leave our vapes alone!” ”@[DrPublicHealth] @truthinitiative @TobaccoFreeKids @XXnews you are running well-funded misinformation campaigns to eliminate the most effective means of smoking cessation…and you sir, scare me far more than any tobacco company.”
Opposition to Flavor Bans and Restrictive Policies	121 (1)	Posts that directly oppose flavor bans or restrictive regulations related to THR products, while excluding general advocacy for flavors that does not explicitly mention bans or policies.	”Flavored e-liquids helped millions quit. The flavor ban hurts those doing this to become healthier.” “@[CongressmanXX] No more taxes/bans on tobacco harm reduction products. Flavored nicotine vaping and other THR products have reduced smoking rates to all-time recorded lows. Stop sending smokers to cancer, copd and death.” ”@[DocCanada]: BAN ON FLAVORS IN VAPING —AN IRRESPONSIBLE AND DOGMATIC DECISION.”
Personal Testimonials and Lived Experience	113 (0.9)	Posts that use first-person narratives to describe a transition from smoking to newer products, emphasizing lived experience and perceived benefits, and it excludes broader claims about safety or harm reduction that lack a personal experiential focus, which are coded under Advocacy for Safer Alternatives.	“I smoked for 40+ years. I am very healthy now because I Quit Smoking & started Vaping. Vaping Is proven to be 95% Safer than Smoking!” ”Just realized it’s been a full year since I’ve had a cigarette. Flavored nicotine vaping is a miracle for former smokers.” “Every survey of adult nicotine ecig users clearly show 80%‐90% prefer flavors other than tobacco or menthol. I for one use strawberry rice pudding to disassociate…I don’t want to smoke anymore, took 14 yrs to find an alternative.”

a THR: tobacco harm reduction.

b Any personally identifiable information was removed or rephrased (eg, ProfTobaccoControl), including handles of individual authors and portions of their content.

##### Theme 1: Advocacy for Safer Alternatives

This theme encompasses the most common pro-THR narrative, promoting newer products as safer alternatives to smoking cigarettes. A total of 7602 (61.3%) posts of pro-THR posts fall in this theme. They position THR as a pragmatic and innovative strategy for reducing smoking-related morbidity and mortality. The discourse often references scientific studies, regulatory recognitions, such as the FDA’s MRTP orders, and endorsements from research institutions like Public Health England and the Royal College of Physicians. A widely circulated claim within this theme is that “vaping is 95% less harmful than smoking,” along with repeated use of language emphasizing reduced risk.

##### Theme 2: Global Models and Comparative Policy Successes

This theme highlights the use of international comparisons to legitimize and promote THR strategies (n=3201, 25.8% posts). Many pro-THR posts mention specific countries, most notably Sweden (n=1294), Japan (n=282), the United Kingdom (n=276), New Zealand (n=233), the United States (n=200), and Australia (n=178). Specific countries are referenced to illustrate how progressive policy frameworks, regulatory support, or public health integration of newer products can lead to lower smoking rates and better health outcomes. Sweden, for instance, is frequently praised for its widespread adoption of snus and near smoke-free status, while the United Kingdom and New Zealand are cited for their supportive THR policies and clinical guidance. In contrast, countries like the United States, Australia, and India are criticized for emphasizing complete cessation or regulatory barriers that allegedly obstruct THR and perpetuate smoking-related harms. These posts present international case studies as real-world evidence of THR’s public health potential. THR advocates (notably many from Australia) use country-level examples to frame THR as a globally validated solution and to influence relevant policies within their regions.

##### Theme 3: Distrust of Public Health Institutions and Suspicion of Financial or Political Influence

This theme reflects pervasive skepticism toward public health institutions, regulatory agencies, and philanthropic organizations engaged in tobacco control, particularly in their stance on THR (1012/, 8.2%). Overall, pro-THR posts frequently tag or mention organizations such as the WHO (n=2015), FDA (n=945), US Centers for Disease Control and Prevention (CDC; n=275), American Lung Association (n=442), American Heart Association (n=145), American Cancer Society (n=99), and Bloomberg Philanthropies (n=298). Of these, many portray these entities as ideologically rigid, scientifically inconsistent, or financially compromised. They argue that such institutions prioritize political agendas, donor-driven interests, or tobacco tax revenue over the adoption of evidence-based THR strategies. Many posts draw historical comparisons, such as the delayed public health response to cigarette harms, to frame current resistance to THR as a continuation of institutional failure. Specific allegations include ignoring modern scientific consensus, protecting revenue streams like the Master Settlement Agreement, or upholding abstinence-only frameworks that disregard pragmatic public health innovations of THR. The tone of these posts is accusatory or frustrated, characterizing opposition to THR as a form of regulatory capture or willful negligence.

##### Theme 4: Endorsement of Industry Efforts for THR

This theme captures posts that mention the industry, including the Big 4 and other manufacturers or retailers of newer products, for their contributions to THR by developing and providing such products (n=158, 1.3%). Although less frequent than other themes, these posts are notable for their attempt to reframe industry actors as contributors to, rather than obstacles in, the future of public health.

These posts in this theme present the industry as a potential partner in advancing smoking cessation and public health goals. Praise is often directed at the industry’s innovation, investment in product safety, and market transitions away from combustible tobacco. While some posts express residual skepticism about corporate motives, they argue that rejecting industry participation outright is counterproductive and rooted in ideological bias rather than public health pragmatism. These posts commonly include claims that the industry is “doing more than public health institutions” to reduce smoking-related harm or that the development of newer products represents meaningful progress. Several posts defend industry-funded research, highlight shifts in product portfolios, or call for public health actors to engage constructively with these developments rather than resist them due to institutional distrust.

##### Theme 5: Defense of Adult Autonomy and Critique of Youth-Centered Regulation

This theme captures intersecting narratives that minimize public health concern over youth vaping while defending the rights of adults to access safer alternatives (n=304, 2.5% posts). Posts in this category argue that the focus on youth has been exaggerated and is often manipulated by institutions to justify restrictive policies, such as flavor bans, tax increases, and marketing limitations. These measures ultimately harm adult smokers attempting to transition away from combustible tobacco. Core to this theme is the belief that THR is not only a matter of public health but also one of civil liberties, consumer rights, and human dignity. Many posts express frustration with what they perceive as paternalistic regulation and advocate for policies that prioritize the needs of adults. Hashtags like #WeVapeWeVote and #StopTheBan frequently appeared in this discourse, reflecting efforts to mobilize adult users and assert resistance against youth-centered regulatory frameworks.

##### Theme 6: Criticism of Anti-THR Narratives as Misinformation

This theme captures sharp critiques of public health narratives and organizations that oppose THR (n=379, 3.1%). Posts under this category characterize anti-THR claims, such as expert warnings, institutional campaigns, or studies highlighting the risks of newer products, as misinformation, fearmongering, or ideologically motivated rhetoric. These messages assert that anti-THR actors are distorting science, spreading baseless claims, or intentionally ignoring evidence to undermine THR. The tone ranges from mocking and sarcastic to highly indignant or accusatory. Posts often amplify pro-THR perspectives by discrediting critics and positioning them as barriers to progress. A defining feature of this theme is its directness, with posts frequently tagging or calling out or even offending specific individuals, researchers, public health agencies, or organizations viewed as opposing THR. These named targets are accused of promoting misleading messages, failing to acknowledge scientific data, or putting lives at risk by resisting access to safer alternatives. In contrast, posts that defend the reduced risk or cessation benefits of newer products are celebrated and framed as honest, evidence-based public health efforts.

##### Theme 7: Opposition to Flavor Bans and Other Restrictive Policies

This theme includes pro-THR posts that express explicit opposition to policies restricting access to flavored e-liquid products particularly (121, 1%). Many posts in this theme argue that such regulations, ranging from flavor bans and taxation to vape mail prohibitions and licensing restrictions, are counterproductive, ideologically motivated, or harmful to public health. THR advocates frequently cite flavored products as essential tools in their own or others’ smoking cessation journeys. These posts often emphasize adult preferences for nontobacco flavors and reject the notion that flavors are designed solely to appeal to youth. Instead, they argue that removing flavored options may drive users back to cigarette smoking, reversing THR gains. Posts within this theme frequently express frustration with public health agencies, such as the FDA or WHO, or local and national governments, positioning flavor bans as violations of consumer autonomy and setbacks to smoking cessation.

##### Theme 8: Personal Testimonials and Lived Experience

This theme captures first-person narratives in which individuals describe their personal success using newer products, most commonly vaping. A total of 113 (0.9%) posts were classified under this theme. These posts typically recount how switching to these alternatives helped them quit smoking, improve their health, or enhance their overall quality of life after struggling with traditional cessation methods. Messages in this theme are grounded in lived experience and emotional reflection, often conveying a sense of relief, gratitude, or empowerment. Many portray THR as a turning point after repeated failures with other approaches, highlighting its value as a practical, real-world solution rather than an abstract policy ideal.

Although this theme sometimes overlaps with broader advocacy narratives found in Theme 1 (Advocacy for Safer Alternatives), it is distinguished by its testimonial tone and emphasis on personal transformation rather than scientific or institutional endorsement. The testimonial format may indicate attempts to humanize the THR debate, framing it as a grassroots movement led by individuals who have directly experienced its benefits.

### Anti-THR Narratives

#### Overview

Of the 3925 posts classified as anti-THR valence, 2208 posts were assigned a single theme while 674 posts had 2 themes. No posts were coded with more than 3 themes. Among anti-THR posts, the most used hashtags included: #WorldNoTobaccoDay (n=844), #Tobacco (n=303), #QuitSmoking (n=257), #NoTobaccoDay (n=189), #NoTobacco (n=187), #CommitToQuit (n=166), #WorldNoTobaccoDay2021 (n=156), #SayNoToTobacco (n=151), #NoSmoking (n=123), and #Health (n=118) (see Table 6).

**Table 6.** Anti-THR^a^ narratives: key themes, inclusion and exclusion criteria, and examples.

Theme	Post, n (%)	Inclusion and exclusion criteria	Representative examples
Calls to Quit and Tobacco-Free Messaging	1955 (49.8)	Posts that explicitly call for individuals to quit all forms of tobacco and nicotine use or endorse tobacco-free goals, often using motivational language or global campaign hashtags, such as #WorldNoTobaccoDay, while excluding posts that focus solely on industry critique or health risks without a direct cessation call.	Say no to tobacco. Be the generation that breaks the chain of addiction. #TobaccoFreeGeneration World No Tobacco Day is observed to encourage people to quit tobacco use. Let’s commit to a healthier future. #CommitToQuit Join us in building a #TobaccoFree world. Quit smoking today for a healthier tomorrow!
Health and Safety Concerns About Newer Products	865 (22)	Posts that highlight health risks of newer products—such as lung injuries, toxic chemical exposure, or long-term illness—often cite scientific or regulatory sources, while excluding posts that center primarily on youth behavior or addiction progression.	Think vaping is harmless? Think again. It’s not just water vapor—there are real health risks. Vaping may seem safer, but it’s linked to lung injuries and toxic chemical exposure. @[Researcher@Harvard] warns: e-cigarettes are not safe. Don’t fall for the marketing.
Youth Protection and Gateway Concerns	384 (9.8)	Posts that focus on tobacco or nicotine use among youth, framing newer products as a gateway to smoking or addiction and calling for protective measures, while excluding posts that discuss general health risks without making youth the central concern.	Flavored vapes are targeting kids. This is a crisis that demands immediate action. Teen vaping is a gateway to lifelong addiction. Ban these products now! @TruthInitiative: We must protect our youth from Big Tobacco’s new tricks.
Support for Comprehensive Bans and Regulatory Action	326 (8.3)	Posts that explicitly support strong policy and legal interventions, such as bans, taxes, or international frameworks like WHO FCTC^b^, while excluding posts that promote cessation at the individual level without referencing regulatory or legal action.	India has banned e-cigarettes, a strong move to protect youth from the new nicotine trap. Countries must enforce stronger regulations on vaping to protect public health. #HealthForAll We support WHO in its fight against tobacco. Comprehensive legislation is essential.
Distrust of the Tobacco Industry and Profit Motives	45 (1.1)	Posts that name and criticize industry actors, such as PMI^c^, BAT^d^, or JUUL, portraying their THR efforts as profit-driven or deceptive, while excluding posts that critique THR products in general without linking concerns to corporate intent or manipulation.	Influencers & freebies: Big Tobacco’s push to hook the next generation. They’re just after profit, not health. @[VapeIndustryWorker] you shouldn’t allow tobacco companies to rebrand vaping as safe. It’s not. Big Tobacco’s ’safer’ alternatives are just their way of keeping you addicted. FCTC5.3 is more important than ever in the extreme marketing war of Philip Morris with cigarettes and alternative dangerous tobacco products
Moral and Ethical Imperatives to End Tobacco Use	26 (0.7)	Posts that frame nicotine use as a moral, ethical, cultural, or religious issue, arguing that tolerating or promoting nicotine violates collective responsibilities to protect public health, while excluding posts that oppose nicotine only on health or regulatory grounds.	World No Tobacco Day is observed around the world every year on 31 May. What WHO is doing to fight the tobacco epidemic, and what people around the world can do to claim their right to health and healthy living and to protect future generations. Join the global movement on World No Tobacco Day! Let’s empower individuals worldwide to say NO to tobacco and YES to a healthier future. Together, we can build a smoke-free world and protect future generations’ well-being.

a THR: Tobacco harm reduction.

b WHO FCTC: World Health Organization’s Framework Convention on Tobacco Control.

c PMI: Philip Morris International.

d BAT: British American Tobacco.

#### Theme 1: Calls to Quit and Tobacco-Free Messaging

This theme represents the most dominant narrative in anti-THR posts, capturing 1955 (49.8%) posts of the anti-THR posts. Posts in this category center on explicit calls for individuals to quit all forms of tobacco and nicotine use, often emphasizing the urgency of cessation as a public health imperative. The messaging is frequently motivational, using language, such as “quit,” “say no,” and “stop now,” and often draws from or reinforces official tobacco control campaigns. Many posts align closely with global initiatives, such as World No Tobacco Day, prominently featuring hashtags like #QuitTobacco, #CommitToQuit, and #WorldNoTobaccoDay. These posts promote cessation as the only legitimate and healthy path forward, contrasting sharply with THR approaches. They often present a tobacco-free future as both a moral ideal and a collective societal goal. Organizations, such as public health agencies, government entities, and non-governmental organizations, were especially active in this theme. Overall, Theme 1 serves as a central pillar of anti-THR discourse by reinforcing complete cessation messaging and institutional public health goals.

#### Theme 2: Health and Safety Concerns About Newer Products

This theme includes posts that highlight general health risks of newer products, such as respiratory complications, toxic chemical exposure, or long-term illness, often citing scientific studies, regulatory advisories, or investigative journalism. Posts often used language like “vaping is not safe,” “toxic,” or “these products harm youth,” and expressed skepticism toward products with THR claims (n=865, 22%).

#### Theme 3: Youth Protection and Gateway Concerns

This theme included posts that focused on tobacco or nicotine use among youth and adolescents, framing vaping as a gateway to smoking or a source of early addiction (n=384, 9.8%). Inclusion was based on the use of youth-centered language (eg, “teen,” “epidemic,” “crisis,” and “protect our kids”) and appeals to parents, educators, or policymakers. Posts frequently referenced teen vaping statistics, school-related concerns, and calls for regulatory action, such as flavor bans.

#### Theme 4: Support for Comprehensive Bans and Regulatory Action

This theme emphasized the importance of strong legal and policy interventions to reduce the use of newer products (n=326, 8.3%). Support was expressed for measures, such as bans on flavored products, higher taxes, plain packaging, and marketing restrictions. Many posts referenced global health governance frameworks, including the WHO FCTC and the United Nations Sustainable Development Goals, particularly those addressing noncommunicable disease prevention. Common phrases included “protect public health,” “tobacco kills,” and “ban nicotine products.” Posts frequently framed regulation as an urgent moral and governmental responsibility.

#### Theme 5: Distrust of the Tobacco Industry and Profit Motives

This theme captured deep skepticism toward the tobacco industry’s involvement in promoting THR (n=45, 1.1%). Posts framed companies, such as PMI, BAT, Altria, and JUUL as driven not by public health goals but by profit and addiction maintenance. Some drew on the industry’s legacy of deception to argue that newer products are part of a rebranding campaign aimed at preserving market dominance and addicting the next generation. Critiques targeted marketing strategies, such as influencer campaigns and flavor promotions, often portraying them as manipulative and harmful. Regulatory references, including Article 5.3 of the WHO FCTC, were invoked to underscore the need to shield public health policy from industry interference.

Representative messages included: “Big Tobacco’s push to hook the next generation. They’re just after profit, not health”; “Big Tobacco’s ‘safer’ alternatives are just their way of keeping you addicted”; and “FCTC5.3 more important than ever in the extreme marketing war of Philip Morris.”

#### Theme 6: Moral and Ethical Imperatives to End Nicotine Use

Closely tied to the previous theme, these posts in this theme framed all forms of nicotine use as a moral issue (n=26, 0.7%). Posts argued that the continued promotion or tolerance of nicotine violates ethical obligations to safeguard public health and community well-being. Messages often invoked collective responsibility, with phrases such as “we must protect future generations” or “no compromise on nicotine.” Some posts carried religious or cultural undertones, asserting that nicotine use is inherently harmful and socially unacceptable.

## Discussion

### Principal Findings

Concerns regarding THR messaging continue within the tobacco control community. Critics argue that the tobacco industry’s THR messaging may undermine comprehensive tobacco control efforts by creating misconceptions about the health risks and cessation efficacy of newer products, interfering with cessation campaigns, enhancing the industry’s reputation, and normalizing tobacco or nicotine use [45,65]. Additionally, these messages frame the industry as a solution provider rather than a root cause of the tobacco epidemic, thereby facilitating entry points for dialogue between industry and policymakers [1,5,66-69]. Such developments may violate the WHO FCTC, which explicitly recommends excluding industry from health policy development [14]. Despite the increasing prominence of THR discourse—both at international forums, such as the FCTC and across social media platforms—empirical studies investigating stakeholder narratives on THR in these digital spaces remain limited. This study contributes to filling that gap by offering a comprehensive analysis of global THR discourse on X, where diverse stakeholders converge and influence public opinion.

Our findings revealed that pro-THR posts made up the majority of the discourse, outnumbering anti-THR and neutral or mixed posts across all years, geographic regions, and most author types. This is consistent with prior research showing the dominance of protobacco or provaping content on social media [35,36,47]. The prevalence of pro-THR messaging can be partly explained by the high volume of content generated by THR advocates, the industry and industry affiliates, and tobacco users. However, a notable finding is that even among groups traditionally seen as skeptical of industry motives—such as health care providers or scientists (716/1629, 44%) and tobacco control advocates (84/364, 23.1%)—pro-THR valence appeared more often than expected. This may reflect optimism about less restrictive policies or the perceived potential of newer products to reduce smoking prevalence. It is also possible that the term “tobacco harm reduction” is more frequently used by individuals or organizations predisposed to support the concept, creating a form of selection bias in discourse. THR opponents may deliberately avoid the term, opting for broader antitobacco or nicotine-free language. For instance, the most frequently used hashtags in anti-THR posts were #WorldNoTobaccoDay, #QuitSmoking, and #NoTobacco, which emphasized cessation and abstinence rather than harm reduction.

Individual and organizational voices with anti-THR perspectives, such as governments, tobacco control organizations, and public health researchers, were less likely to contribute to THR discourse compared to their pro-THR counterparts in our data. In this context, while pro-THR narratives are highly visible on social media, substantial debate remains regarding the risks, benefits, and appropriate regulation of newer products. A recent study also found that industry-related users like vape advocates represent most interactions related to tobacco regulations on social media platforms; therefore, they are more likely to be amplified and reach a larger audience [70]. As demonstrated in previous research, sustained exposure to dominant message valence on social media can create perceived social norms, shaping attitudes and behaviors [28,29]. This underscores the need to amplify evidence-based, independent voices from governments, tobacco control organizations, and public health researchers—and to strengthen their collaboration in the online THR space to ensure diverse perspectives are represented until more conclusive evidence on THR emerges.

Our comparative thematic analysis revealed clear distinctions between pro- and anti-THR narratives. Pro-THR posts emphasized scientific rationalism and personal agency, frequently citing research findings, scientists, regulatory decisions, and international policy models, alongside personal testimonies of successful smoking cessation. Hashtags, such as #HarmReduction, #VapingSavesLives, and #SaferNicotine framed THR as an innovative and compassionate solution. Many of these messages also advocated for consumer rights, adult autonomy, and equitable access to alternatives. In line with these narratives, some researchers and public health experts also advocated for the potential role of THR in reducing smoking-related harm.

In contrast, anti-THR narratives—aligned with a public health and prevention-oriented perspective—relied more on moral, absolutist, and precautionary messaging. These posts focused on ending all forms of tobacco and nicotine use, portraying THR as a smokescreen for continued addiction. Common hashtags included #CommitToQuit and #TobaccoFreeGeneration, reflecting calls for complete cessation. Prominent themes included appeals to protect youth and future generations, skepticism toward newer products, and strong warnings about deceptive marketing by the tobacco industry. Anti-THR messages often aligned with broader anti-industry sentiment and expressed support for global health authorities, such as the WHO.

The volume of THR discourse surged during key events, such as regulatory announcements, product authorizations or bans, and global campaigns like World No Tobacco Day. These peaks were marked by sharp increases in both pro- and anti-THR posts, indicating that regulatory milestones serve as catalysts for public engagement and polarization. Pro-THR advocates framed regulatory approvals as scientific justifications of THR, whereas anti-THR voices interpreted the same actions as threats to youth safety and public health. Notably, during instances of restrictive regulation or product bans, pro-THR posts shifted their narrative to emphasize adult autonomy and the need for safer alternatives. Conversely, anti-THR posts reinforced support for such policies, citing industry manipulation, youth exploitation, and the potential harms of normalizing nicotine use. World No Tobacco Day consistently intensified this dichotomy, with pro-THR advocates accusing the WHO of ignoring evidence-based harm reduction and anti-THR advocates endorsing the organization’s approaches.

Our analysis also revealed regional disparities. Pro-THR sentiment was most concentrated in high-income countries, particularly Oceania, Europe, and North America. Posts from Australia and the United Kingdom frequently criticized what they viewed as outdated or prohibitionist models of tobacco control. In contrast, anti-THR sentiment was more common in lower-middle-income countries, such as Pakistan and Kenya, where newer products are less accessible and tobacco control efforts may focus more on cessation and abstinence. These disparities raise concerns about equity and the global diffusion of THR strategies. In countries with limited healthcare infrastructure or regulatory capacity, the adoption of THR messaging, especially when driven by industry, may lead to confusion or exacerbate health disparities. Pro-THR messaging may resonate more strongly in regions where newer products are accessible, the tobacco industry has an extensive advocacy network, and public discourse surrounding newer products or policies is more prominent.

For instance, the US FDA’s MRTP-related decisions and the surrounding discourse can be misinterpreted and influence lower-income countries, especially when the industry uses these decisions to promote expensive newer products and their perceived potential. Similarly, a study analyzed news coverage of the FDA’s MRTP order for IQOS in middle- and low-income countries and found that nearly half of the reports contained inaccuracies regarding the authorization or the product itself, emphasizing economic narratives over scientific accuracy [71].

The growing presence of industry in the THR discourse is significant. This aligns with previous research, which found that much of the social media content related to tobacco policy, particularly on X, is more indicative of industry-affiliated users than of genuine public sentiment. The study cautions researchers to be mindful of the potential for artificial amplification of industry-driven public relations and marketing efforts on social media platforms [70]. In our data, nearly 40% of pro-THR posts referenced specific names or categories of new products, and over 15% contained direct marketing attempts. The trend of increasing presence of the Big 4, tobacco manufacturers and retailers, and THR advocates should be importantly noted, as it underscores the industry’s evolving digital strategy in amplifying THR discourse on social media. These tactics mirror established marketing approaches on social media [72-75].

This study has several limitations that should be acknowledged. First, we used content analysis to examine THR discourse on X. While this method is well suited for identifying patterns and themes across large datasets, it is subject to coder interpretation and potential bias, which may affect the accuracy and reliability of thematic categorization. Second, our study was conceptually focused on THR discourse as a strategic frame and did not include product-specific terms in the initial keyword query. Although product names were systematically identified and coded during the textual analysis, it is possible that some posts discussing THR solely through product names (while omitting the term “tobacco”) were not captured by the initial search. Future research should expand search terms to include brand- and product-level keywords to maximize coverage of THR discourse.

Third, our classification of THR advocates is limited to their affiliations and does not capture the full range of potential motives within this group. Specifically, we cannot fully determine whether THR accounts are directly or indirectly linked to industry influence, for example, through organizational funding, professional ties, or the circulation of industry-produced materials. At the same time, independent actors, such as grassroots consumer advocates, academics, and public health care professionals may promote THR outside of direct industry affiliation. This limitation highlights the need for caution in interpreting pro-THR content as homogeneously motivated or organized. Attributing the prevalence of pro-THR narratives solely to industry influence would oversimplify the dynamics of the discourse. It also underscores the importance of distinguishing between independent advocacy voices and industry-affiliated actors, as both play distinct roles in shaping the conversation around THR. This distinction is critical for understanding the broader social, scientific, and policy debates surrounding harm reduction.

Fourth, the dataset was restricted to English-language posts, which constrains global representation and contributes to the high concentration of posts from North America and other high-income countries in our sample. THR discourse in non-English-speaking contexts, particularly in regions with high smoking prevalence or distinctive regulatory environments, may reflect culturally specific perspectives that are not captured here. Future research should expand multilingual data collection to better represent the diversity of global discourse. Our analysis was restricted to the July 2019–December 2023 period, which excludes pre-EVALI (E-cigarette or Vaping Product Use–Associated Lung Injury) discourse and therefore limits comparisons across pre- and postcrisis narratives. Fifth, the dataset was drawn exclusively from X, which does not fully represent the broader social media environment or public discourse. User demographics, engagement styles, and discursive practices vary considerably across platforms. Incorporating cross-platform data would provide a more comprehensive view of public perceptions and industry influence. Finally, we relied on geotagging provided by Quid, which estimates country-level origin based on available metadata. Posts without valid geographic information were excluded, reducing the completeness of location-based findings.

In conclusion, this study offers a comprehensive examination of global discourse surrounding THR on X, identifying dominant themes, stakeholder participation, and shifts in message valence over time and across regions. Our findings show that pro-THR valence dominates online conversations, particularly in high-income countries where newer products are more widely available and industry presence is substantial. This dynamic has the potential to influence and potentially mislead THR discourse and policy development in low-income countries as the industry expands the sale of newer products. Additionally, we identified notable and potentially concerning trends between 2020 and 2023, including increases in overall THR post volume (from 3578 to over 5057), posts by THR advocates (from 1325 to 3014), mentions of newer products (from 1653 to 1731), and marketing attempts within THR discourse (from 439 to 1210). These shifts present new challenges for tobacco regulation and public health advocacy in the digital space, particularly in countering the dominance of industry perspectives on THR and their influence on public understanding and policy development.

Our findings have both scholarly and practical implications. From a research standpoint, our findings highlight the growing presence of THR discourse on X in recent years, aligning with scholarly perspectives that view social media platforms as key spaces where public discourse on tobacco-related policies unfolds, thereby offering valuable data for examining timely conversations around tobacco policy [48]. Future studies may build on this work by examining the long-term effects of dominant social media narratives on consumer behavior, regulatory outcomes, and public trust in health authorities and other THR stakeholders. From a policy perspective, the disproportionate presence of industry-affiliated and pro-THR messaging on digital platforms underscores the need for greater visibility and responsiveness from independent experts, policymakers, and advocacy organizations, and their stronger collaboration across nations and regions. Public health campaigns must actively engage in these digital spaces—not only to correct misinformation but also to offer clear, evidence-based guidance on THR and related products for both consumers and policymakers.

Most newer products come with explicit and implicit THR claims [2]; however, scientific evidence regarding their relative health risks compared to conventional cigarettes remains inconclusive. Their effectiveness in aiding smoking cessation also lacks robust validation, even for those products that have received MRTP authorization [76-79]. Our findings point to an urgent need for communication strategies that are responsive to both the persuasive strength of pro-THR narratives and the underrepresentation of anti-THR messaging.

Our research indicates that online THR discourse is increasingly shaped by a dynamic interplay of forces beyond industry and government engagement, including THR advocates who call for stronger scientific evidence on traditional tobacco control measures, as well as broader shifts in societal attitudes toward nicotine and tobacco alternatives. To ensure informed decision-making, tobacco control authorities should recognize this plurality of drivers, attend to emerging needs, amplify their presence in online spaces, and engage directly with contested narratives around THR. Monitoring social media trends, identifying emerging frames and actors, and integrating these insights into tobacco control policy will be critical for countering industry influence and promoting equitable health outcomes.

### Conclusion

This study provides a comprehensive analysis of global discourse on THR across social media, highlighting the dominance of pro-THR narratives, particularly in high-income countries where newer products are widely available and industry influence is substantial. The increasing visibility of pro-THR content, coupled with the growth of advocacy and marketing messages between 2020 and 2023, underscores how industry and affiliated voices continue to shape public conversations about THR. Such dynamics risk skewing public understanding and policy debates, especially in low- and middle-income countries where independent public health communication remains limited. Our findings also emphasize the need for tobacco control stakeholders to actively engage in digital spaces to counter misinformation, amplify evidence-based perspectives, and ensure balanced representation in global THR discourse.

53

## Supplementary material

10.2196/77676 Checklist 1 STROBE checklist.

## Abbreviations.

BAT: British American Tobacco
CDC: US Centers for Disease Control and Prevention
e-cigarettes: electronic cigarettes
ENDS: electronic nicotine delivery systems
EVALI: E-cigarette or Vaping Product Use–Associated Lung Injury
FDA: US Food and Drug Administration
JTI: Japan Tobacco International
MRTP: modified risk tobacco product
PMI: Philip Morris International
RQ: research question
THR: tobacco harm reduction
WHO: World Health Organization
WHO FCTC: World Health Organization’s Framework Convention on Tobacco Control
