# Supplementary material for: Message Valence, Industry Influence, and Stakeholder Narratives in Global Conversations on Tobacco Harm Reduction: Content Analysis
Source: J Med Internet Res. 2025 Nov 3;27:e77676. doi: 10.2196/77676 (PMC12582543; doi:10.2196/77676)
Supplement: Checklist 1 [file jmir-v27-e77676-s001.docx]

# **Appendix 2. STROBE Checklist – Cross-sectional study.**

| **Item** | **Recommendation** | **Reported on page/section** |
| --- | --- | --- |
| 1a | Indicate study design in title/abstract | Title, Abstract |
| 1b | Provide informative/structured abstract | Abstract |
| 2 | Background/rationale | Introduction |
| 3 | Objectives | Introduction (RQ1–RQ6) |
| 4 | Study design | Methods |
| 5 | Setting | Methods (Timeframe: July 2019–Dec 2023) |
| 6a | Participants/data sources | Methods (X posts via Quid API) |
| 6b | Eligibility criteria | Methods (English-language; irrelevant/duplicate removed) |
| 7 | Variables | Methods (valence, author type, geography, products, marketing) |
| 8 | Data sources/measurement | Methods (coding, text mining, Krippendorff’s alpha) |
| 9 | Bias | Methods (manual validation, clarified industry vs. advocacy) |
| 10 | Study size | Methods (17,361 posts final sample) |
| 11 | Quantitative variables | Methods (percentages, χ² tests) |
| 12a | Statistical methods | Methods (descriptive stats, chi-square tests) |
| 12b-e | Subgroups/interactions/missing data | Methods, Results (geographic/economic groups; missing location acknowledged) |
| 12f | Sensitivity analyses | Discussion (keyword scope limitation) |
| 13a | Participants | Results (flow: 167,867 → 17,361 posts) |
| 13b | Non-participation | Results (ads, duplicates removed) |
| 14a | Descriptive data | Results (posts by year, region, author type) |
| 14b | Missing data | Results (country-level missing reported) |
| 15 | Outcome data | Results (valence distributions, author types, etc.) |
| 16a | Main results | Results (numerical outcomes, χ² tests) |
| 16b | Category boundaries | Results (economic classification, author types) |
| 16c | Absolute risks | Not applicable (observational discourse analysis) |
| 17 | Other analyses | Results (thematic analysis of narratives) |
| 18 | Key results | Discussion (summary by RQ) |
| 19 | Limitations | Discussion (search scope, English-only, platform limits, timeframe) |
| 20 | Interpretation | Discussion (balanced, neutral, alternative explanations) |
| 21 | Generalizability | Discussion (global sample; English-only bias noted) |
| 22 | Funding | Funding Statement |
